# Influence of carbon-based organic fertilizer on soil microorganisms and vegetable growth in Sb-As co-contaminated soil: a synergistic mechanism of promotion and detoxification

**DOI:** 10.3389/fpls.2026.1823671

**Published:** 2026-08-03

**Authors:** Shuwen Zhao, Xuanshuo Wang, Dan Wu, Yuling Yang, Yumin Yang, Yuxian Shangguan, Jining Li

**Affiliations:** 1 Institute of Agricultural Resources and Environment, Sichuan Academy of Agricultural Sciences, Chengdu, China; 2 Chengdu University of Technology, Chengdu, China; 3 National-Regional Joint Engineering Research Center for Soil Pollution Control and Remediation in South China, Guangdong Key Laboratory of Integrated Agro-environmental Pollution Control and Management, Institute of Eco-environmental and Soil Sciences, Guangdong Academy of Sciences, Guangzhou, Guangdong, China; 4 School of Environment, Nanjing Normal University, Nanjing, Jiangsu, China

**Keywords:** antimony, arsenic, co-contamination, rhizosphere, soil remediation

## Abstract

**Background:**

Antimony (Sb) and arsenic (As) co-contamination threatens crop production and food safety. Carbon-based organic fertilizers may simultaneously improve soil fertility, regulate microbial communities, and reduce metal(loid) mobility, but their performance in Sb-As co-contaminated vegetable systems remains unclear.

**Methods:**

A pot experiment with pak choi was conducted to compare different carbon-based amendments, including magnesium sulfate-loaded biochar-based organic fertilizer (CBOF-M). Soil chemistry, pore-water elements, enzyme activities, rhizosphere microbial communities, plant growth, and elemental accumulation were evaluated.

**Results:**

Carbonaceous amendments reduced Sb mobility but increased As solubility. CBOF-M increased soil carbon, produced the highest plant biomass, reduced Sb translocation to edible tissues, and enriched Bacteroidota and Gemmatimonadota. Structural equation modeling identified an indirect microbiome-mediated pathway and a direct enzyme-mediated nutrient-cycling pathway.

**Conclusion:**

Magnesium sulfate-loaded biochar-based organic fertilizer produced a promotion-detoxification synergy, although As mobilization remains a key constraint requiring formulation-specific control.

## Graphical Abstract

**Figure:**
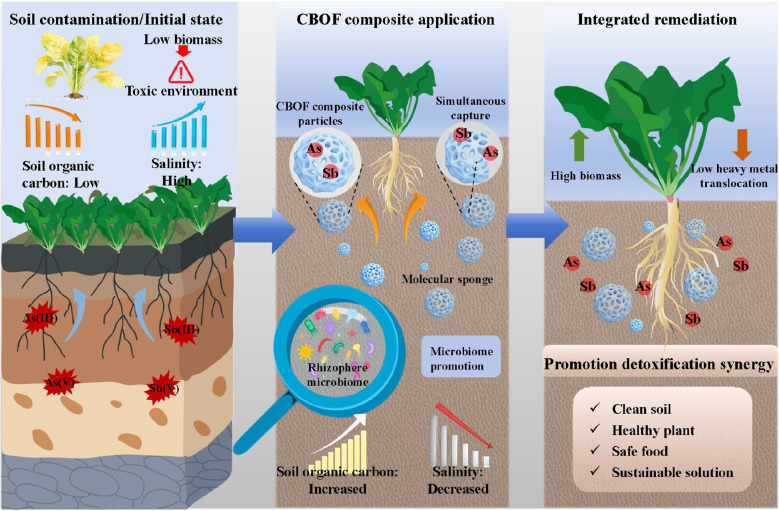
The abstract should ideally be structured according to the IMRaD format (Introduction, Methods, Results and Discussion). Provide a structured abstract if possible. If your article has been copyedited by us, please provide the updated abstract based on this version.

## Highlights

- MgSO_4_-loaded biochar exhibited a unique dual functionality, enhancing soil organic carbon sequestration while simultaneously reducing electrical conductivity and salinity.
- Carbon-based amendments exhibited a competitive effect on metalloid mobility, effectively immobilizing Sb but paradoxically increasing As solubilization in soil pore water.
- The MgSO_4_-loaded biochar enabled a promotion-detoxification synergy, significantly enhancing Pak Choi biomass while suppressing Sb translocation to edible tissues.
- Structural equation modeling revealed that the promotion-detoxification synergy operates via dual pathways: indirect microbiome-mediated effects and direct biochemical stimulation.

## 1 Introduction

Antimony (Sb) and arsenic (As), toxic metalloids designated as priority pollutants (Li et al., 2026; Tu et al., 2026), pose a severe environmental threat globally, especially in mining regions. As the world’s leading Sb producer, China accounts for approximately 60% of global output, with concentrated mining in Hunan, Guangxi, and Guizhou provinces (U.S. Geological Survey, 2025). These activities release substantial amounts of Sb and geochemically co-associated As into ecosystems (Filella et al., 2002), exacerbating soil contamination through mismanaged wastes. This pollution compromises soil health and enters the food chain—particularly via leafy vegetables—posing significant risks to human health (Gai et al., 2026). Both non-essential elements inhibit crucial plant processes like germination, root development, and photosynthesis, leading to reduced growth and crop vitality (Wu et al., 2019). Therefore, developing effective remediation strategies is urgent. Carbon-based organic fertilizer presents a promising solution, offering potential for the simultaneous immobilization of Sb and As, soil quality improvement, and the safe production of vegetables, addressing a key need in sustainable agriculture.

Conventional soil remediation often employs single amendments, which are typically effective for specific heavy metals but less suitable for co-contamination scenarios like Sb and As, and may cause secondary issues such as soil acidification or nutrient loss (Xie et al., 2022). Therefore, integrated amendments have gained attention. Carbon-based organic fertilizer (CBOF) represents an advanced composite material that synergistically combines the high adsorption and stabilization capacity of biochar with the nutrient supply of organic fertilizer. Its porous structure not only facilitates the immobilization of contaminants, reducing their bioavailability, but also improves soil structure and fertility (Wang et al., 2022). This dual functionality supports the simultaneous goals of contaminant stabilization and plant growth promotion. Recent studies have demonstrated that the co-application of biochar and bioorganic fertilizer can improve rhizosphere soil microecology, reduce soil electrical conductivity, enhance nutrient availability, and promote plant growth in saline-alkaline soils (Gu et al., 2023). Similarly, biochar and organic fertilizer application has been reported to improve soil nutrient status, increase enzyme activities, reshape bacterial community composition, and enhance plant productivity in Cd-contaminated soils (Liu et al., 2024). From the perspective of contaminant immobilization, biochar-based organic fertilizers can reduce the weak acid-extractable fractions of heavy metals and promote their transformation into more stable residual forms, thereby decreasing metal accumulation in plant tissues (Qu et al., 2025). Long-term field evidence further indicates that biochar and organic fertilizer inputs, especially their combined application, can increase the abundance of functional microbial groups involved in C, N, P, and S cycling and improve agroecosystem multifunctionality (Hu et al., 2024). These findings suggest that CBOFs may be particularly suitable for contaminated agricultural soils because they couple contaminant stabilization, nutrient supply, and microbial regulation.

Soil microorganisms are fundamental to ecosystem functions and are highly sensitive to amendments (Banerjee et al., 2018; Geisseler et al., 2017). Composite amendments, particularly those combining biochar and organic matter, are known to improve the soil microbial habitat (Zhou et al., 2019). Biochar provides a porous substrate and can adjust soil conditions, while organic fertilizer supplies abundant organic carbon and nutrients. Together, they can enhance microbial diversity, abundance, and activity (Awasthi et al., 2020). CBOF leverages this synergy to create a favorable environment for microbial communities (Hong et al., 2022). This revitalized microbial environment likely contributes to nutrient cycling, soil health, and may participate in the biogeochemical transformation of Sb and As, thereby supporting more sustainable remediation. However, the specific mechanisms through which CBOF reshapes the microbial community and influences plant-microbe interactions in Sb and As co-contaminated soils remain poorly understood and require further investigation. However, most existing studies have focused on single-metal contamination, especially Cd, Pb, Cu, and Zn, or on soil improvement under salinity stress. Relatively little is known about how engineered CBOFs regulate the coupled behavior of Sb and As in co-contaminated agricultural soils. In particular, the links among CBOF-induced changes in soil chemistry, rhizosphere microbial community structure, soil enzyme activities, and vegetable growth under Sb-As stress remain unclear.

Based on this background, the present study focuses on the Sb-As co-contaminated soil surrounding the Qinglong antimony smelter in Guizhou Province. Through pot experiments, the effects of different carbon-based organic fertilizers, particularly magnesium sulfate-loaded biochar, were investigated with respect to soil physicochemical properties, heavy metal speciation transformation, soil enzyme activities, and microbial community structure. The regulatory role of these amendments on the growth and heavy metal accumulation of pak choi (Brassica rapa var. glabra Regel) was also analyzed. This research aims to elucidate the mechanisms by which carbon-based organic fertilizers synergistically alleviate Sb-As stress through integrated pathways of “soil chemistry-microbial ecology-plant growth and elemental partitioning”. The findings are expected to provide a theoretical foundation and technical support for the application and promotion of carbon-based materials in the safe utilization of co-contaminated agricultural fields.

## 2 Materials and methods

### 2.1 Soil collection and preparation

Topsoil (0–20 cm) was collected from farmland adjacent to a historic antimony smelter in Qinglong County, Guizhou Province, China (25°33′-26°11′N, 105°01′-105°25′E), representing a typical site of long-term Sb and As co-contamination. Following collection, the soil was air-dried at room temperature, manually homogenized, and sieved through a 2-mm mesh to remove stones and plant debris. The initial physicochemical properties of the soil are presented in **Table 1**. The prepared soil was stored in airtight containers at 4°C until use.

**Table 1:** Effects of carbon-based organic fertilizer on chemical properties of root zone soil.

Treatment	O	T1	T2	T3	T4	T5	T6
pH	8.07 ± 0.05a	8.04 ± 0.14a	8.08 ± 0.14a	8.03 ± 0.10a	7.98 ± 0.07a	8.06 ± 0.05a	8.01 ± 0.09a
OM(g kg-1)	139.74 ± 6.82a	138.82 ± 6.50a	140.24 ± 8.17a	144.31 ± 5.77a	139.51 ± 3.25a	140.09 ± 5.94a	136.77 ± 6.37a
TN(g kg-1)	27.46 ± 5.04ab	32.76 ± 1.31ab	31.75 ± 1.13ab	33.66 ± 0.58a	27.98 ± 0.80ab	31.06 ± 1.40ab	26.02 ± 4.33b
P2O5(g kg-1)	0.52 ± 0.02b	0.62 ± 0.00a	0.59 ± 0.01ab	0.59 ± 0.01ab	0.59 ± 0.02ab	0.57 ± 0.05ab	0.59 ± 0.03ab
K2O(g kg-1)	5.28 ± 0.12b	5.56 ± 0.15ab	5.50 ± 0.21ab	5.57 ± 0.07ab	5.67 ± 0.04a	5.50 ± 0.10ab	5.61 ± 0.10ab
NH4-N(mg kg-1)	11 ± 3a	13 ± 4a	7 ± 1a	10 ± 2a	10 ± 3a	8 ± 1a	7 ± 2a
NO3-N(mg kg-1)	35 ± 1a	17 ± 2b	21 ± 2b	14 ± 4b	18 ± 5b	30 ± 2a	15 ± 3b
TOC(g kg-1)	160.77 ± 2.53ab	153.10 ± 5.30b	157.20 ± 5.70ab	153.03 ± 5.15b	156.20 ± 4.10ab	161.53 ± 4.46ab	166.57 ± 5.43a
TC(g kg-1)	214.27 ± 6.27ab	209.97 ± 6.68ab	216.40 ± 13.55ab	224.63 ± 3.25a	200.60 ± 4.67b	219.07 ± 1.78ab	226.40 ± 5.66a
EC(mS/cm)	0.357 ± 0.069a	0.259 ± 0.097a	0.279 ± 0.111a	0.282 ± 0.041a	0.257 ± 0.065a	0.221 ± 0.044a	0.186 ± 0.058a

### 2.2 Plant material and preparation

Seeds of Pak Choi (*Brassica rapa* var. *glabra* Regel) were obtained from a commercial supplier. Pak choi (Brassica rapa var. glabra Regel) was selected as the test crop because it is a widely cultivated leafy vegetable in China, has a short growth cycle and high sensitivity to soil environmental stress, and its edible leaves provide a direct pathway for human exposure to metal(loid)s. Compared with many other Brassica crops, pak choi is particularly suitable for pot-based remediation studies because its rapid biomass response and edible shoot tissues allow simultaneous evaluation of plant growth promotion and food safety risks under Sb-As co-contaminated conditions. To ensure sterility, seeds were surface-disinfected by immersion in 10% (v/v) H_2_O_2_ for 10 minutes, followed by thorough rinsing with deionized water. Subsequently, seeds were germinated on moist filter paper in Petri dishes and incubated in the dark at 25°C for 48 hours. Uniformly germinated seeds with a radicle length of 2–3 mm were selected for transplantation.

### 2.3 Experimental design and pot setup

A completely randomized pot experiment was conducted in a controlled greenhouse from May to July 2025. Polyethylene pots (20 cm upper diameter × 18 cm height) were each filled with 2.5 kg of the prepared soil. A total of seven treatments were established, each with six replicates: (1) control (CK), unamended soil; (2) biochar (BC, T1), soil amended with 2% (w/w) wheat straw biochar; (3) hydrothermal carbon (HTC, T2), soil amended with 2% (w/w) hydrothermal carbon; (4) hydrothermal carbon liquid (HTCL, T3), soil amended with 2% (w/w) hydrothermal carbon liquid; (5) commercial carbon-based organic fertilizer (CBOF-C, T4), soil amended with 2% (w/w) commercial CBOF; (6) ferrous sulfate-modified biochar (BC+Fe, T5), soil amended with 2% (w/w) FeSO_4-_loaded biochar; and (7) modified CBOF (CBOF-M, T6), soil amended with 2% (w/w) magnesium sulfate-loaded biochar-based organic fertilizer. Briefly, wheat straw was washed with deionized water, oven-dried at 65°C, ground, and sieved through a 20-mesh sieve. The prepared wheat straw powder was pyrolyzed at 450°C for 2 h under a N_2_ atmosphere, with a heating rate of 10°C min^-^¹, to obtain the pristine wheat straw biochar. For the FeSO_4-_modified biochar, the pristine biochar was impregnated with FeSO_4_ solution at a solid-to-liquid ratio of 1 g:10 mL, shaken at 300 rpm for 12 h, aged for 12 h, filtered, and oven-dried at 70°C. For the MgSO_4-_loaded biochar, the pristine biochar was impregnated with 0.20 mol L^-^¹ MgSO_4_·7H_2_O solution using the same solid-to-liquid ratio of 1 g:10 mL, shaken at 300 rpm for 12 h, aged for 12 h, filtered, dried at 70°C, ground, and sieved to 0.15-1.00 mm. The CBOF-M was prepared by mixing 60 parts MgSO_4-_loaded biochar, 35 parts decomposed chicken-manure organic fertilizer, and 5 parts hydrothermal-char liquor dry matter. The moisture content was adjusted to 20%-30%, followed by thorough mixing for 20–40 min and low-temperature drying or granulation to obtain 1–5 mm particles with a final moisture content below 15%.The use of biochar and biochar-based organic fertilizers was based on previous studies demonstrating their effectiveness in improving soil properties and immobilizing heavy metals or metalloids in contaminated soils (Ahmad et al., 2014; Rajapaksha et al., 2016; Qu et al., 2025). Hydrothermal carbon and hydrothermal carbon liquid were included because hydrothermal carbonization products can provide soluble organic carbon and nutrients and can modify soil chemical conditions (Sun et al., 2014). Fe-modified biochar was selected because iron oxides and Fe-loaded biochar have strong affinity for arsenic species through surface complexation and co-precipitation (Wu et al., 2018; Beiyuan et al., 2023). Mg-modified biochar-based organic fertilizer was designed to integrate carbon supply, nutrient regulation, and metal(loid) stabilization functions, following the rationale of engineered biochar amendments for contaminated soils (Rajapaksha et al., 2016; Liang et al., 2021). The amendment rate of 2% (w/w) was selected according to previous pot experiments and remediation studies using biochar or biochar-based amendments in contaminated soils. All amendments were thoroughly homogenized with the soil. The amendment rate of 2% (w/w) was selected based on previous pot experiments and preliminary screening, which indicated that this rate can effectively improve soil chemical conditions and reduce metal(loid) bioavailability while avoiding excessive changes in salinity or pH (Ahmad et al., 2014; Rajapaksha et al., 2016; Li et al., 2024). Post-amendment, pots underwent a two-week equilibration period under controlled environmental conditions (25/20°C day/night, 60% field water capacity). Then, three pre-germinated seedlings were transplanted per pot and later thinned to one plant. During the 60-day growth period, plants were watered with deionized water as needed and maintained under a 14-hour photoperiod without additional fertilizer or pesticide application.

### 2.4 Plant and soil sampling

#### 2.4.1 Plant harvest and processing

At harvest (60 days after transplantation), plant height, leaf number, and fresh weight were recorded. Plants were carefully uprooted. Roots were separated from shoots and rinsed to remove soil. A subsample of fresh root and shoot tissue was flash-frozen in liquid nitrogen and stored at -80°C for biochemical analysis. The remainder was oven-dried at 75°C for 72 hours to determine dry weight, then ground to a fine powder. For elemental analysis, approximately 0.5 g of powdered plant tissue was digested with concentrated HNO_3_ and H_2_O_2_ using a gradient heating program. This digestion procedure was adapted from commonly used acid digestion protocols for plant and environmental samples, and the resulting digests were analyzed by inductively coupled plasma optical emission spectrometry (ICP-OES) according to standard instrumental procedures (USEPA, 1996a, 1996b).

#### 2.4.2 Soil sampling and analysis

Rhizosphere soil was collected at harvest. One portion was immediately frozen on dry ice for microbial community analysis. Another portion was air-dried, then oven-dried at 102°C for 72 hours, ground, and sieved through a 100-mesh sieve (<0.149 mm) for analysis of standard soil chemical properties (pH, EC, OM, TC, DOC, mineral N, TN, available P and K) and for Sb/As chemical speciation using a sequential extraction procedure. Soil pH, EC, OM, TC, TOC, mineral nitrogen, TN, available P, and available K were determined according to standard soil agrochemical analysis methods (Bao, 2000). Soil Sb and As chemical fractions were determined using a sequential extraction procedure modified from Tessier et al. (1979).Because the soil used in the pot experiment was air-dried, homogenized, sieved, and packed into pots at the same soil mass, initial differences in soil physical structure among treatments were minimized. Therefore, this study focused mainly on soil chemical and biological indicators related to metal(loid) mobility, nutrient cycling, microbial responses, and plant growth. Soil physical properties, including bulk density, porosity, and aggregate stability, were not measured and should be further evaluated under field conditions.

#### 2.4.3 Pore water analysis

Two weeks before final harvest, rhizosphere pore water was collected using suction-cup samplers (EasySensor SFS), filtered, and stored frozen for soluble element analysis by ICP-OES. Fresh roots were subjected to the dithionite-citrate-bicarbonate (DCB) extraction procedure. Rhizosphere pore water was collected using suction-cup samplers and filtered prior to ICP-OES analysis.

### 2.5 Microbial community analysis

Samples for microbial community analysis—including rhizosphere soil, root endophytes, and leaf endophytes—were collected and processed as follows. For leaf endophyte analysis, two to three fully expanded, healthy mature leaves from the middle part of each pak choi plant were collected at harvest. Leaves with visible mechanical damage, chlorosis, or disease symptoms were avoided. Fresh leaves (2–3 pieces) and root segments (3–5 segments, ~5 cm each) were surface-sterilized by sequential rinsing with distilled water (30 s), 70% ethanol (2 min), 2.5% NaClO (5 min), and 70% ethanol again (30 s), followed by three washes with sterile water. Rhizosphere soil was collected by gently shaking off loose soil and then brushing the tightly adhered soil from the roots with a sterile brush. All samples were immediately frozen in liquid nitrogen and stored at −80°C until further analysis. Microbial DNA extraction and high-throughput sequencing of bacterial (16S rRNA gene) and fungal (ITS) communities were conducted by Guangdong Magigene Biotechnology Co., Ltd. (Guangzhou, China) using Illumina NovaSeq platforms according to the manufacturer’s standard protocols. Sequence processing and taxonomic assignment were performed following commonly used microbial community analysis workflows, with reference to the SILVA ribosomal RNA database for bacterial classification (Quast et al., 2013).

### 2.6 Statistical analysis

All statistical analyses were performed in R. Differences among treatments were tested using one-way ANOVA followed by Tukey’s HSD test or Kruskal-Wallis test when assumptions of normality and homogeneity of variance were not met. Microbial alpha diversity and Bray-Curtis dissimilarity were calculated using the vegan package (Oksanen et al., 2022). Differences in microbial community composition were visualized by PCoA and tested using PERMANOVA (Anderson, 2001). Differentially abundant microbial taxa were identified using LEfSe (Segata et al., 2011). Structural equation modeling was conducted using the lavaan package, and model fit was evaluated using χ² test, CFI, and RMSEA (Rosseel, 2012).”

## 3 Results and discussion

### 3.1 Alterations in rhizosphere soil physicochemical properties induced by carbon-based organic fertilizer

The systematic application of carbon-based amendments precipitated profound and differentiated alterations in the physicochemical matrix of the Sb-As co-contaminated rhizosphere soil, with treatment-specific outcomes underscoring the functional specialization of each material (**Table 1**). While core parameters such as soil pH and total organic matter content remained statistically invariant across the treatment spectrum, significant and targeted modulations were observed within critical nutrient cycles and edaphic stress indicators. Specifically, pH, OM, EC, and NH_4-_N did not differ significantly among the control and amendment treatments. The stability of soil pH was likely related to the strong buffering capacity of the original weakly alkaline soil, which weakened the short-term alkalizing or acidifying effects of the added amendments. The absence of a significant change in OM may be explained by the high background organic matter content and the relatively short duration of the pot experiment, because total OM represents a relatively stable and heterogeneous pool that usually responds more slowly than labile carbon fractions (Lehmann and Kleber, 2015). Although EC showed a decreasing trend, especially under the CBOF-M treatment, the differences were not statistically significant because soluble ions are highly heterogeneous in rhizosphere microsites and are strongly affected by root uptake, ion exchange, and amendment adsorption processes. Therefore, the EC response was interpreted as a non-significant decreasing tendency rather than a statistically confirmed reduction. Similarly, NH_4-_N is a highly dynamic nitrogen form that can be rapidly transformed through nitrification, immobilized by microorganisms, adsorbed by soil colloids or biochar surfaces, or absorbed by plant roots, resulting in large temporal and spatial variability at harvest (Booth et al., 2005; Kuzyakov and Blagodatskaya, 2015). These factors collectively explain why pH, OM, EC, and NH_4-_N did not show significant differences among treatments in **Table 1**. A clear paradigm of functional complementarity emerged among the amendments. The hydrothermal carbon liquid treatment (T3) demonstrated a pronounced efficacy in enhancing the total nitrogen (TN) pool, achieving a 22.58% increase relative to the control. This enhancement is likely mechanistically linked to the direct introduction of soluble, plant-available nitrogen species within the liquid amendment and/or the stimulation of microbial nitrogen mineralization processes, a phenomenon documented with organic inputs in contaminated soils (Gao et al., 2021). In contrast, the availability of phosphorus (P_2_O_5_) was uniquely and significantly elevated in the T1 treatment, which lacked added biochar, suggesting a potential suppression or competitive immobilization of P induced by certain carbonaceous matrices.

The most integrative performance was clearly demonstrated by the MgSO_4-_loaded biochar (CBOF-M, T6). This engineered composite increased both total organic carbon (TOC) and total carbon (TC) pools and showed the lowest mean EC value among all treatments. However, because EC differences were not statistically significant, this result should be interpreted as a decreasing tendency rather than direct evidence of significant salinity mitigation. This positions CBOF-M as a multi-functional amendment capable of addressing both carbon sequestration goals and salt stress alleviation in degraded lands (Yang et al., 2021). The mechanistic basis for this functional differentiation is rooted in the distinct physicochemical architectures of the amendments. These findings are generally consistent with previous studies showing that biochar-organic fertilizer combinations can improve soil nutrient retention, organic carbon input, and microbial habitat conditions more effectively than single amendments (Gu et al., 2023; Hu et al., 2024). For example, Gu et al. (2023) reported that the co-application of biochar and bioorganic fertilizer improved rhizosphere soil microecology and plant growth in saline-alkaline soil, while Hu et al. (2024) found that biochar and organic fertilizer inputs enhanced soil functional microbial abundance and agroecosystem multifunctionality. However, the limited change in soil pH observed in the present study differs from many previous reports in which biochar markedly increased soil pH (Ahmad et al., 2014; Rajapaksha et al., 2016). This discrepancy is likely attributable to the weakly alkaline background of the tested soil and its strong buffering capacity, which weakened the short-term alkalizing effect of the amendments. The notable reduction in EC, particularly associated with modified biochars like CBOF-M, aligns with their documented role as geochemical sponges, capable of adsorbing soluble ions or participating in ion-exchange reactions that directly ameliorate osmotic stress (Li et al., 2020a).

Concurrently, the significant augmentation of stable soil carbon fractions under CBOF-M treatment underscores its role transcending mere contaminant immobilization; it acts as a direct, recalcitrant input into the soil organic matter continuum, contributing to long-term carbon stabilization (Li et al., 2018). Interestingly, the stability of soil pH across all treatments, despite the intrinsic alkalinity characteristic of many biochars, reveals the moderating influence of the soil’s inherent buffering capacity and the specific chemical interactions introduced by modifications such as MgSO_4_ loading. This buffering effect, preventing drastic pH swings, has been observed in other systems employing modified biochars and is critical for maintaining microbial habitat suitability (Qayyum et al., 2021). This context-dependent response powerfully illustrates a cornerstone principle in environmental remediation: the impact of any amendment is not an intrinsic property but arises from a dynamic interplay between the amendment’s specific characteristics and the complex, pre-existing biogeochemistry of the soil environment (Elkhlifi et al., 2021). Therefore, the empirical evidence presented here strongly advocates for a precision-remediation approach, where amendments are selectively matched to the specific nutritional deficiencies and physicochemical constraints of the target co-contaminated soil. The relatively high variability observed in some parameters, particularly EC and NH_4_^+^-N, likely reflects the heterogeneous nature of rhizosphere soil microsites and the dynamic nature of these labile soil properties, which are influenced by both amendment characteristics and plant-root mediated processes.

Values are mean ± standard error (n = 3). Different lowercase letters indicate significant differences among treatments at P < 0.05 according to one-way ANOVA followed by Tukey’s HSD test. Treatment abbreviations: CK or O, unamended control; BC or T1, biochar; HTC or T2, hydrothermal carbon; HTCL or T3, hydrothermal carbon liquid; CBOF-C or T4, commercial carbon-based organic fertilizer; BC+Fe or T5, ferrous sulfate-modified biochar; CBOF-M or T6, magnesium sulfate-loaded biochar-based organic fertilizer. Soil property abbreviations: OM, organic matter; TN, total nitrogen; P_2_O_5_, available phosphorus expressed as P_2_O_5_; K_2_O, available potassium expressed as K_2_O; NH_4-_N, ammonium nitrogen; NO_3_-N, nitrate nitrogen; TOC, total organic carbon; TC, total carbon; EC, electrical conductivity.

### 3.2 Modulation of elemental accumulation in soil pore water

**Figure 1:**
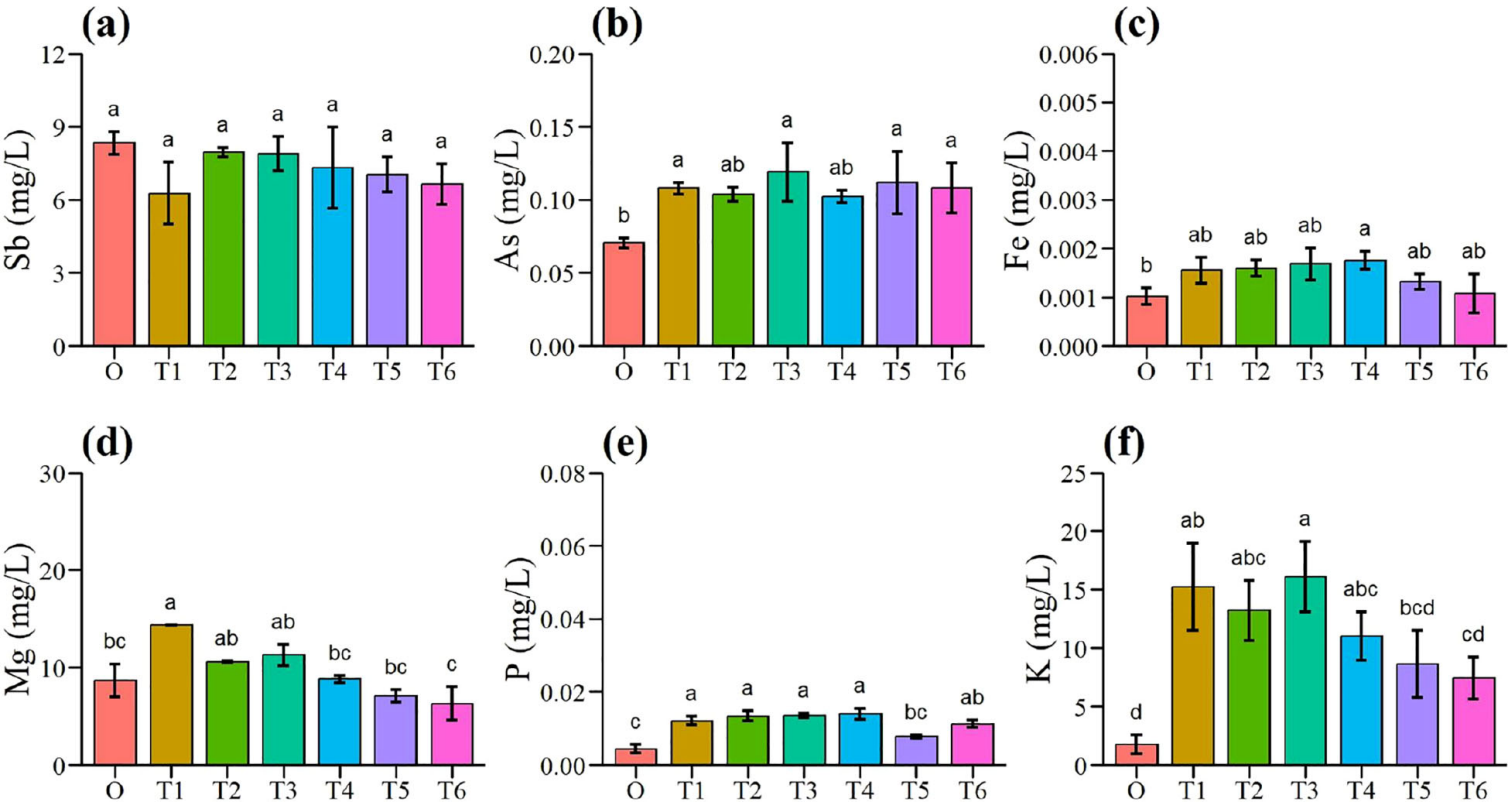
Effects of carbon-based organic fertilizers on the concentrations of Sb, As, and nutrients in soil pore water. Values are presented as mean ± standard error. Different lowercase letters above the bars indicate significant differences among treatments at P < 0.05 according to one-way ANOVA followed by Tukey’s HSD test; bars sharing the same letter are not significantly different.

The application of carbon-based amendments substantially altered the elemental composition of soil pore water, reflecting their differential effects on the bioavailability and mobility of both toxic and nutrient elements. All amendment treatments significantly reduced pore-water Sb concentrations relative to the unamended control (**Figure 1**), with the greatest reduction (24.68%) observed in treatment T1. This finding is consistent with the well-documented high affinity of carbonaceous materials for Sb oxyanions through surface complexation and electrostatic interactions (Li et al., 2024), confirming the efficacy of these amendments in sequestering Sb and limiting its hydrological transport.

In marked contrast, the solubility of As increased across all amendment treatments, with the most pronounced elevation (68.98%) recorded for the hydrothermal carbon liquid (T3). This divergent behavior between Sb and As underscores a fundamental challenge in remediating co-contaminated systems: competitive adsorption and ligand-induced mobilization. At the weakly alkaline pH of the tested soil, the contrasting responses of Sb and As may be related to their different aqueous speciation and surface-binding behavior. Sb is commonly present as Sb(V), mainly as Sb(OH)_6_^-^ under alkaline conditions, and this species can be immobilized through complexation with Fe/Al/Mg-associated surfaces, mineral phases, and oxygen-containing functional groups on biochar. In contrast, As may occur as arsenate oxyanions and is more susceptible to competitive desorption by phosphate, dissolved organic matter, and other organic ligands released from the amendments. These competing ligands can occupy sorption sites on Fe oxides and soil mineral surfaces, thereby enhancing As release into pore water (Redman et al., 2002; Bauer and Blodau, 2006; Li et al., 2024). Therefore, the same carbon-based amendment may immobilize Sb while mobilizing As, particularly in weakly alkaline co-contaminated soils where ligand exchange and competitive adsorption are pronounced. The reduction in pore-water Sb after carbon-based amendment application is consistent with previous studies reporting that biochar and modified biochar can immobilize metal(loid)s through surface complexation, electrostatic attraction, mineral precipitation, and interactions with oxygen-containing functional groups (Ahmad et al., 2014; Rajapaksha et al., 2016). In particular, Li et al. (2024) showed that pristine and Fe-functionalized biochar could remediate As-Sb co-contaminated mining soil, although the immobilization efficiency depended strongly on biochar modification and soil geochemical conditions. In contrast, the increase in As solubility observed in our study is not fully consistent with studies using Fe-functionalized biochar for As immobilization. This difference may be explained by the release of dissolved organic matter and competing anions from some carbonaceous amendments. Dissolved organic matter can compete with arsenate for sorption sites on Fe oxides and mineral surfaces, thereby enhancing As desorption and mobilization (Redman et al., 2002; Bauer and Blodau, 2006). Therefore, the contrasting responses of Sb and As in this study highlight that amendments effective for Sb immobilization may not necessarily stabilize As under the same soil conditions. Amendments rich in soluble organic compounds or phosphate can displace adsorbed As from soil mineral surfaces or form aqueous complexes, thereby enhancing As release into solution—a phenomenon extensively documented in soils co-contaminated with arsenic and organic amendments (Li et al., 2020b).

The pore water analysis also revealed substantial shifts in essential nutrient dynamics. Treatment T3 triggered an extraordinary increase in K concentration (+828.36%), attributable to the direct solubilization and release of K from the hydrothermal carbon matrix. Concurrently, pristine biochar (T4) elevated Fe and P levels, underscoring its capacity to modulate the solubility of key nutrients through pH-mediated dissolution and competitive desorption mechanisms.

The conventional compound fertilizer treatment further increased the solubility of both Sb and As, serving as a cautionary example of how standard agricultural practices can inadvertently exacerbate contaminant mobility in polluted soils (Li et al., 2024). This observation highlights a critical trade-off between agronomic productivity and environmental safety when conventional fertilizers are applied to metalloid-contaminated soils.

Collectively, these results demonstrate that while carbon-based amendments are highly effective for Sb immobilization, their tendency to mobilize As poses a significant limitation that must be addressed through tailored amendment design. The contrasting behaviors of Sb and As observed here reflect their distinct aqueous speciation, surface complexation preferences, and competitive interactions with co-occurring anions, emphasizing the need for composite amendments capable of simultaneously stabilizing both metalloids in co-contaminated environments.

### 3.3 Response of rhizosphere soil enzymatic activities

**Figure 2:**
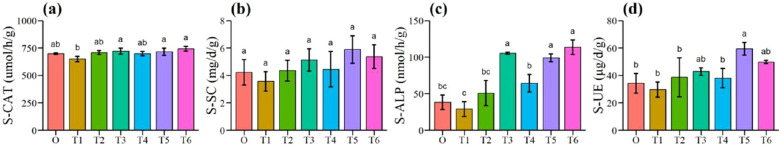
Effects of carbon-based organic fertilizers on the activities of CAT **(A)**, SC **(B)**, ALP **(C)**, and UE **(D)** in rhizosphere soil. Values are presented as mean ± standard error. Different lowercase letters above the bars indicate significant differences among treatments at P < 0.05 according to one-way ANOVA followed by Tukey’s HSD test; bars sharing the same letter are not significantly different.

Soil extracellular enzymes, serving as sensitive and integrative proxies for microbial metabolic investment and soil ecosystem function, provided a detailed biological fingerprint of how the amendments reconfigured soil biochemical processes (**Figure 2**). These enzymes, integral to nutrient mineralization and stress response, are widely recognized as robust indicators of soil health and microbial community adaptation (Aponte et al., 2020; Daughtridge et al., 2021). The amendment-induced changes in enzyme activities observed in this study are broadly consistent with previous reports showing that biochar and organic fertilizer can regulate soil enzymatic processes by altering substrate availability, nutrient status, pH, and microbial community composition (Wang et al., 2015; Hu et al., 2024). The strong increase in alkaline phosphatase activity under T3, T5, and T6 agrees with studies indicating that organic amendments can stimulate phosphorus cycling by increasing microbial demand for P and promoting phosphatase-producing microorganisms. However, the divergent response of urease and catalase among treatments also indicates that enzyme responses are not universally positive. Similar treatment-specific enzyme responses have been reported in amended or contaminated soils, where enzyme activities depend on the balance among nutrient input, metal toxicity, soil pH, and microbial adaptation. Thus, the present results support the view that CBOFs regulate soil biochemical functioning in a material-specific rather than uniformly stimulatory manner. The experiment revealed a landscape of highly treatment-specific enzymatic responses that reflected the distinct chemical properties of each amendment.

Alkaline phosphatase (S-ALP) activity exhibited the most pronounced response, with activities in the T3, T5, and T6 treatments increasing by 175.86% to 196.67% (**Figure 2**). This dramatic activation showed a direct positive correlation with the measured increases in available soil phosphorus reported in Section 3.1. This finding reinforces the established ecological principle that microbial communities invest in phosphatase production in response to phosphorus demand, making S-ALP a reliable bio-indicator of phosphorus cycling status (Kizilkaya and Hepsen, 2004; Tang et al., 2020; Behera et al., 2017).

Urease (S-UE) activity, which governs the hydrolysis of urea and thus serves as a key gatekeeper in the nitrogen cycle, displayed a more complex and divergent pattern. It was significantly stimulated in treatment T2 but markedly suppressed in T3 and T6. This differential response suggests that the various amendments can either facilitate or interfere with the microbial processes and substrate availability governing nitrogen transformation, depending on their specific chemical composition and the resulting soil microenvironment.

Sucrase (S-SC) activity, involved in carbon cycling, remained largely unperturbed across treatments, indicating that the amendments did not fundamentally alter the microbial processing of simpler carbohydrates under these experimental conditions. In contrast, catalase (S-CAT) activity—an enzyme crucial for degrading reactive oxygen species (e.g., H_2_O_2_) and mitigating oxidative stress—showed variable responses. Its suppression in treatment T1 and enhancement in others underscores its high sensitivity to changes in soil redox potential and overall abiotic stress, solidifying its role as a key indicator of soil oxidative status (Yang et al., 2016).

The inhibition of certain hydrolase activities by specific treatments, such as the suppression of urease in T3 and T6 and the reduction of catalase in T1, demonstrates that non-optimized amendments can potentially disrupt soil microbial metabolic networks and enzyme synthesis pathways (Kizilkaya and Hepsen, 2004; Liu et al., 2018). These results collectively indicate that while amendments like the hydrothermal carbon liquid (T3) and MgSO_4-_loaded biochar (T6) were potent modulators of the soil enzymome, their effects are highly context-dependent. The observed enzymatic responses are governed by the specific chemistry of each amendment and its interaction with native soil properties, particularly pH and nutrient status, which ultimately determine the direction and magnitude of changes in soil biochemical processes.

### 3.4 Effects on Pak Choi growth and elemental partitioning

**Figure 3:**
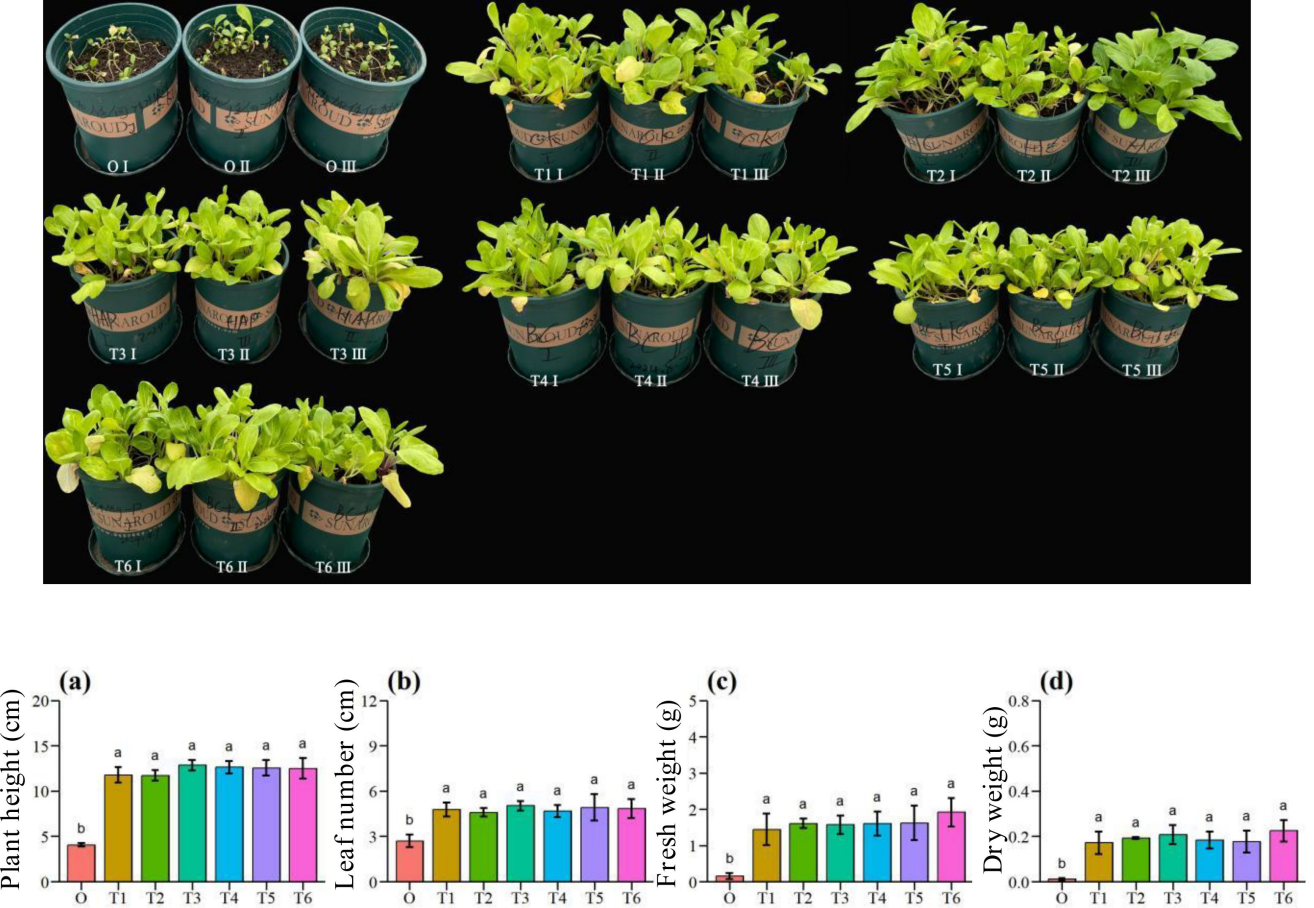
Effects of carbon-based organic fertilizers on plant height **(A)**, leaf number **(B)**, fresh weight **(C)** and dry weight **(D)** of Pak Choi. Values are presented as mean ± standard error. Different lowercase letters above the bars indicate significant differences among treatments at P < 0.05 according to one-way ANOVA followed by Tukey’s HSD test; bars sharing the same letter are not significantly different.

The application of carbon-based organic fertilizers (CBOFs) elicited a profound dual response: dramatic promotion of plant growth coupled with complex reconfiguration of elemental uptake and partitioning within the Pak Choi-soil system. All CBOF treatments uniformly and significantly enhanced every measured biometric parameter—plant height, leaf number, fresh weight, and dry weight—relative to the unamended control (**Figure 3**). Representative photographs of pak choi before treatment and at harvest are shown in **Figure 3**. The visual differences among treatments were consistent with the quantitative biomass data, showing that CBOF treatments improved plant growth compared with the unamended control. The hydrothermal carbon liquid (T3) excelled in promoting vegetative structural growth, while the MgSO_4_-loaded biochar (T6) achieved the highest absolute biomass yields. This “promotion-detoxification” synergy, wherein growth stimulation coincides with contaminant mitigation, represents a hallmark of advanced engineered amendments and aligns with findings that functionalized biochar composites can outperform their pristine counterparts by concurrently improving nutrient supply and alleviating metal stress (Zama et al., 2018).This result is consistent with previous studies showing that biochar-organic fertilizer amendments can improve crop growth under contaminated or degraded soil conditions. Liu et al. (2024) reported that the combined application of biochar and organic fertilizer improved plant productivity and quality in Cd-contaminated soil by reshaping bacterial communities and improving soil nutrient status. Similarly, Qu et al. (2025) found that biochar-based organic fertilizers promoted the transformation of heavy metals from labile fractions to more stable fractions and reduced metal accumulation in plant tissues. Compared with these studies, the present work extends the application of CBOFs from conventional heavy metals such as Cd, Pb, Cu, and Zn to the more challenging Sb-As co-contaminated system. Nevertheless, the increased As solubility observed in pore water suggests that growth promotion and contaminant stabilization may not always occur simultaneously for all metal(loid)s. This difference emphasizes the importance of designing amendment formulations that can balance nutrient supply with the selective immobilization of both Sb and As.

**Figure 4:**
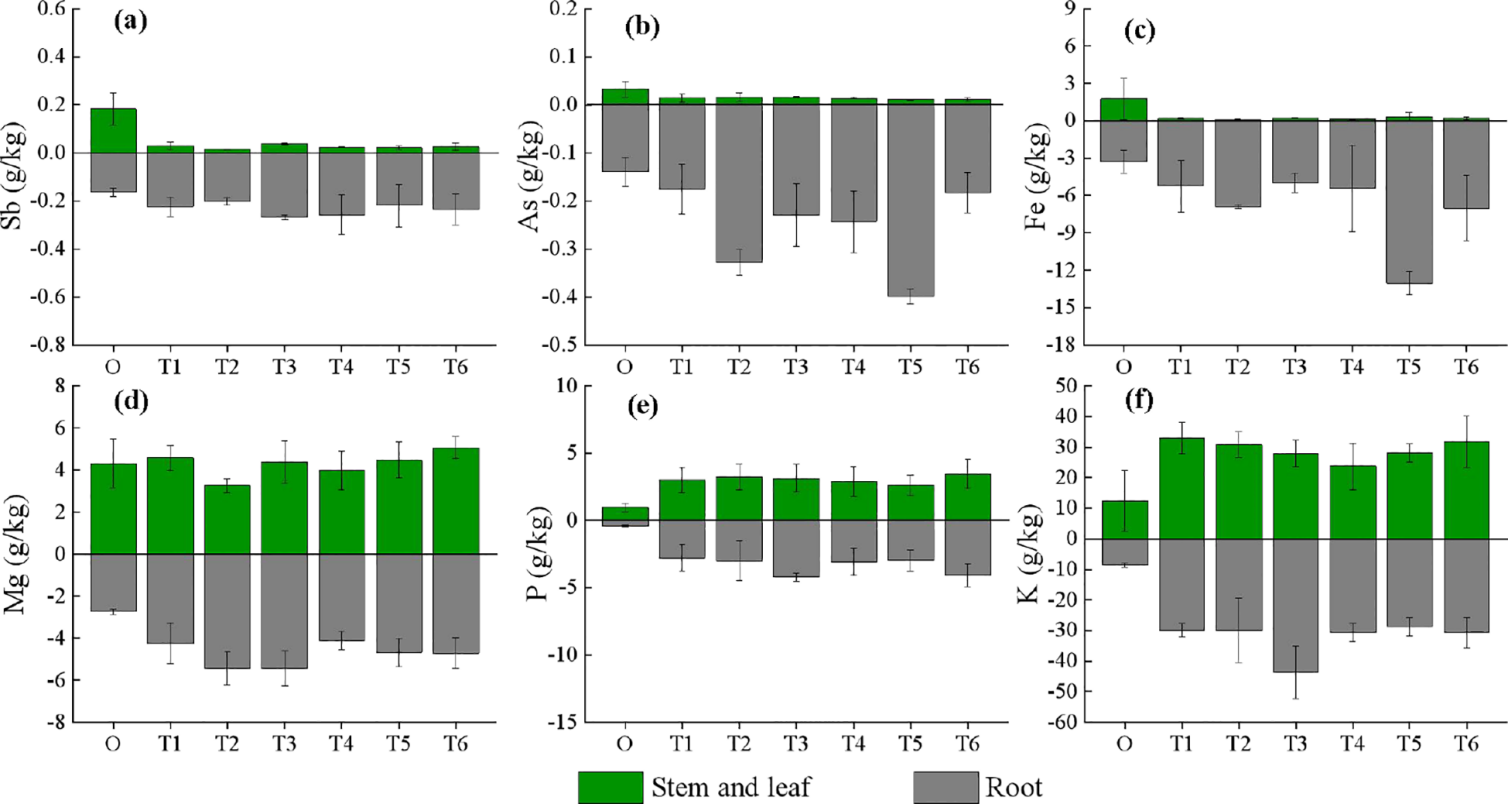
Concentrations of Sb **(A)**, As **(B)**, Fe **(C)**, Mg **(D)**, P **(E)**, and K **(F)** in the roots and stem-leaf tissues of pak choi under different amendment treatments.

The translocation and compartmentalization of toxic metalloids revealed intricate, treatment-dependent mechanisms. CBOFs were notably effective in impeding the vascular translocation of Sb from roots to shoots, with treatment T2 reducing shoot Sb concentration by 91.81%. Concurrently, these amendments promoted the sequestration of As within root tissues, thereby depleting its concentration in the edible aerial shoots—a highly favorable outcome for agricultural safety and food security (**Figure 4**). The contrasting behavior between Sb and As observed here reflects their distinct biogeochemical behaviors: Sb immobilization likely proceeds through surface complexation with biochar functional groups, while As retention in roots may involve precipitation with Fe plaque or sequestration in vacuolar compartments.

Paralleling these contaminant dynamics, the CBOFs substantially improved the plant’s nutritional status. Treatments such as T3 led to extraordinary increases in the root content of essential macronutrients, with phosphorus increasing by 925.31% and potassium by 405.22%. This nutrient enrichment represents a primary mechanism through which biochar-based amendments mitigate abiotic stress and promote growth in suboptimal soils, as adequate P and K are vital for energy metabolism, osmoregulation, and overall plant vigor (Yuan et al., 2025). The enhanced nutrient availability likely results from both direct nutrient supply from the amendments and indirect effects on soil physicochemical properties that increase nutrient solubility and root uptake efficiency.

The differential responses among treatments underscore the importance of amendment composition in determining outcomes. The MgSO_4-_loaded biochar (T6) demonstrated particular efficacy in balancing growth promotion with contaminant restriction, suggesting that targeted surface modifications can enhance the selective immobilization of toxic elements while maintaining or improving nutrient supply. In contrast, treatments lacking biochar or with different compositional matrices showed varying capacities for achieving this balance, highlighting that the specific chemical and structural properties of each amendment govern its ultimate performance.

Collectively, these results demonstrate that strategically formulated CBOFs can effectively address the dual challenges of phytotoxicity and nutrient limitation in co-contaminated soils. By simultaneously acting as soil conditioners, nutrient sources, and targeted immobilizing agents, these amendments offer a viable pathway for the sustainable utilization of metalloid co-contaminated agricultural land while ensuring the safety of food crops.

### 3.5 Restructuring of the rhizosphere microbial community at the phylum level

**Figure 5:**
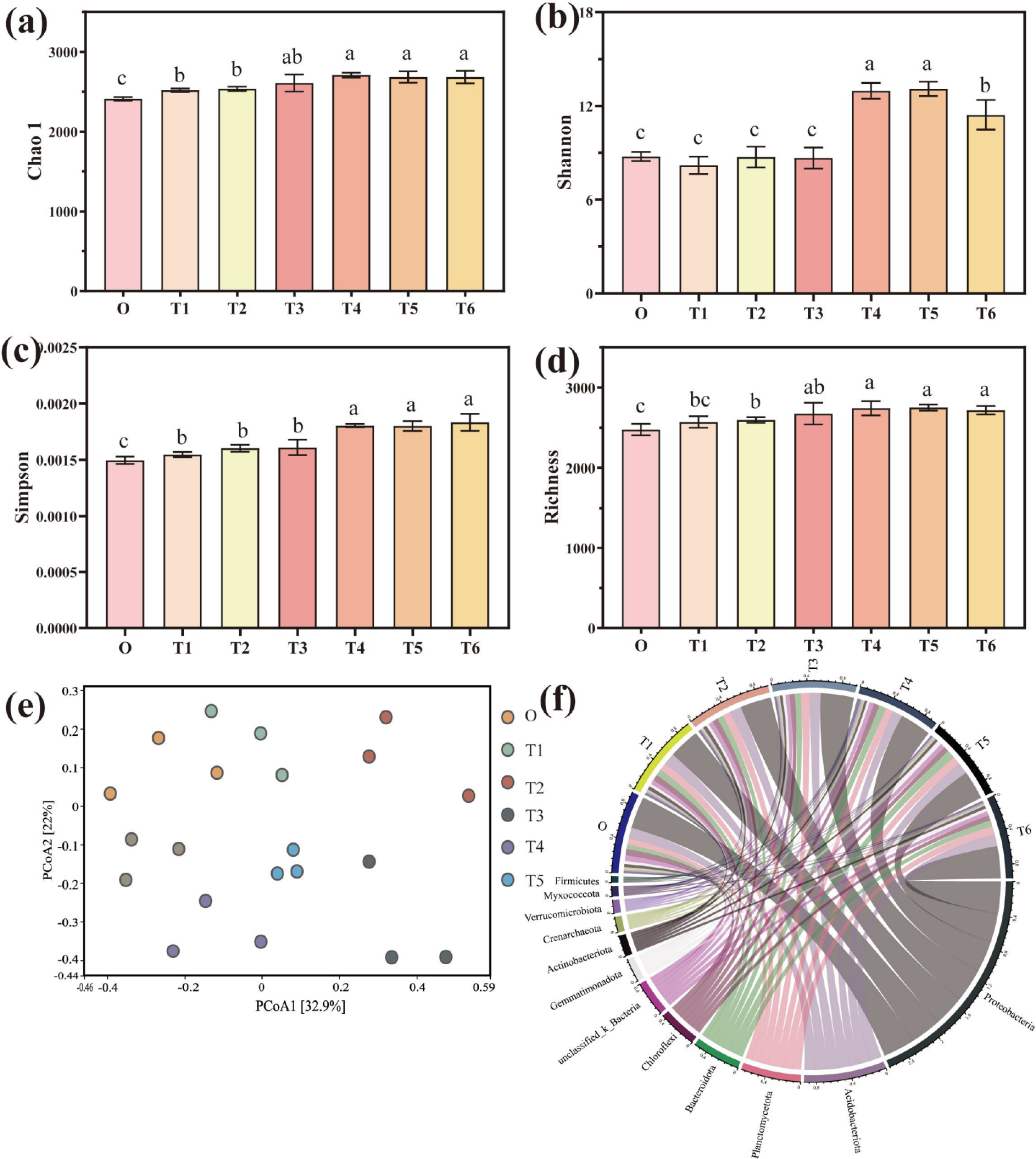
Differences in microorganisms in soil samples. **(A)** Diversity indexes for bacteria(Chao1). **(B)** Diversity indexes for bacteria (Simpson). **(C)** Diversity indexes for bacteria (Shannon). **(D)** Diversity indexes for bacteria (Riches). **(E)** PCoA for bacteria in various regions, with PERMANOVA (Adonis) showing regional differences in bacterial communities. **(F)** The line graphs of bacterial phyla after different treatments. Values are presented as mean ± standard error. Different lowercase letters above the bars indicate significant differences among treatments at P < 0.05 according to one-way ANOVA followed by Tukey’s HSD test; bars sharing the same letter are not significantly different.

The application of CBOFs induced significant and reproducible restructuring of the rhizosphere soil microbial community at the phylum level, reflecting a biological recalibration to the amended soil environment. Across all treatments, the community maintained a consistent core dominated by Proteobacteria (34.70–40.19%), a phylum recognized for its metabolic versatility and prevalence in both contaminated and agricultural soils (**Figure 5**). This was followed in relative abundance by Acidobacteriota, Planctomycetota, Chloroflexi, Bacteroidota, and Gemmatimonadota. The most pronounced treatment effect was observed for the MgSO_4-_loaded biochar-based organic fertilizer, CBOF-M (T6), which drove the most substantial enrichments, increasing the relative abundance of Bacteroidota by 83.26% and Gemmatimonadota by 44.79% compared with CK. The enrichment of Bacteroidota and Gemmatimonadota is consistent with previous studies showing that biochar and organic fertilizer can reshape rhizosphere microbial communities by increasing carbon availability, nutrient supply, and habitat heterogeneity (Gu et al., 2023; Hu et al., 2024). Bacteroidota are often associated with the decomposition of complex organic matter and rapid utilization of labile carbon sources, while Gemmatimonadota have been linked to improved soil nutrient conditions and adaptation to environmental stress. However, the magnitude and direction of microbial shifts differ among studies. For example, some studies reported stronger enrichment of Proteobacteria or Actinobacteriota after biochar addition, which may be related to differences in soil pH, amendment type, crop species, contamination level, and experimental duration. Therefore, the microbial response observed here should be interpreted as a site- and material-specific restructuring of the rhizosphere microbiome rather than a universal microbial succession pattern.

This microbial reorganization represents a direct biological response to the new habitat template created by the amendments. The significant enrichment of Bacteroidota—a phylum often associated with the degradation of complex organic polymers and considered copiotrophic (thriving in resource-rich conditions)—points directly to the increased availability of labile organic carbon and nutrients supplied by the organic fertilizer component and the biochar surfaces. The co-enrichment of Gemmatimonadota, a phylum frequently linked to improved soil nutrient conditions and drier environments, further supports the concept of an overall ameliorated soil habitat. The persistent dominance of Proteobacteria reaffirms its ecological resilience and adaptability, playing a core functional role across the stress gradient (Rampelotto et al., 2013). These taxonomic shifts align with the established paradigm that biochar amendments act as ecosystem engineers, modifying key drivers of microbial community assembly such as pH, pore architecture, nutrient availability, and contaminant toxicity (Igalavithana et al., 2019).

Therefore, the CBOF amendments, and particularly the BC+Mg-P composite, did not merely cause stochastic microbial variation but selectively reshaped the foundational structure of the rhizosphere microbiome. This shift toward a community enriched in phyla such as Bacteroidota and Gemmatimonadota indicates a transition from a stressed, oligotrophic state to a more robust, copiotrophic state. The newly configured microbiome is poised for enhanced functional capacity in organic matter decomposition and nutrient cycling, representing a crucial biological mechanism that links the previously documented improvements in soil physicochemical properties to the ultimate enhancement of plant growth and health in the contaminated environment.

### 3.6 Integrated stabilization and remediation mechanisms

**Figure 6:**
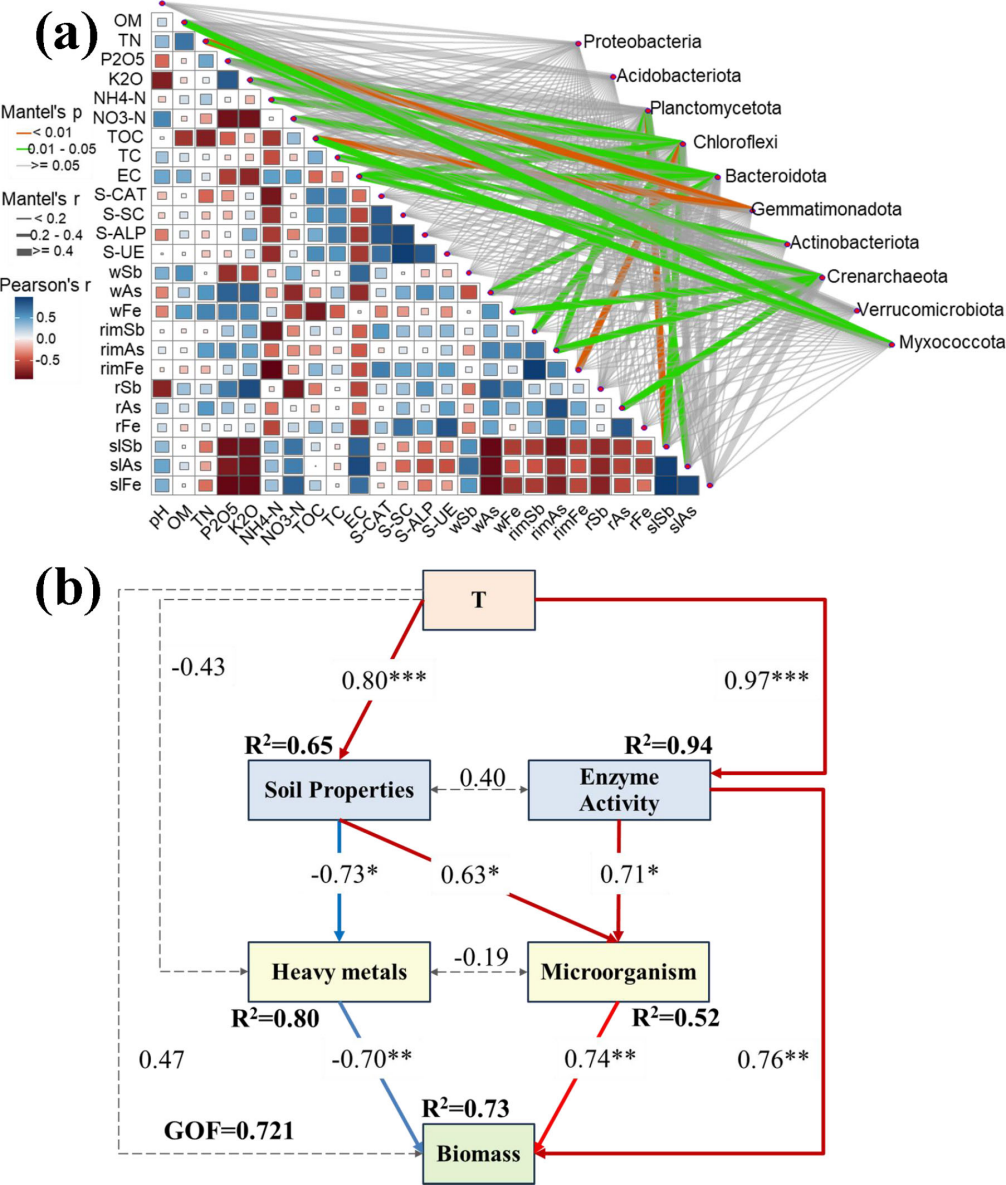
Linking soil properties to plant status via the microbiome: **(A)** Mantel test and **(B)** Structural Equation Model (SEM).

The remediation of Sb–As co-contaminated soil by carbon-based organic fertilizers is governed by an integrated, sequential mechanism that synergistically couples direct physicochemical stabilization with indirect, biologically amplified processes. Statistical validation through Mantel test analyses revealed robust and significant correlations between amendment-altered soil variables (e.g., water-soluble As, EC, available K) and the specific enrichment of microbial taxa such as Planctomycetota and Bacteroidota (**Figure 6A**). Crucially, Structural Equation Modeling (SEM) provided quantitative validation of the causal pathways underlying these relationships (**Figure 6B**). The model explained 78% of the variance in plant biomass and revealed two distinct mechanistic routes: (i) an indirect pathway (path coefficient = 0.64, P < 0.001), wherein biochar-induced improvements in soil physicochemical properties (pH, available nutrients, reduced EC) fostered a beneficial microbial community (enriched Bacteroidota and Gemmatimonadota), which subsequently promoted plant growth; and (ii) a direct pathway (path coefficient = 0.28, P < 0.05), whereby biochar rapidly stimulated soil enzyme activities (particularly alkaline phosphatase and catalase), leading to enhanced nutrient mineralization and stress alleviation independent of microbial community restructuring. These SEM results provide causal evidence for the proposed “promotion-detoxification” synergy, demonstrating that the remediation efficacy arises from both microbiome-mediated indirect effects and direct biochemical stimulation by the amendments. This integrated mechanism is supported by previous studies showing that biochar-based amendments can simultaneously affect soil chemistry, enzyme activity, microbial community structure, and plant performance. For example, Li et al. (2024) reported that pristine and Fe-functionalized biochar altered As and Sb bioavailability, soil enzyme activities, and bacterial communities in As-Sb co-contaminated mining soil. Hu et al. (2024) further demonstrated that biochar and organic fertilizer inputs improved agroecosystem multifunctionality by increasing functional microbial groups involved in C, N, P, and S cycling. Compared with these studies, our SEM analysis further links amendment-induced soil chemical changes, enzyme responses, microbial enrichment, and pak choi biomass into a unified promotion-detoxification framework. This comparison indicates that the novelty of the present study lies not only in testing MgSO_4-_ loaded CBOF in Sb-As co-contaminated soil, but also in quantitatively separating the indirect microbiome-mediated pathway from the direct enzyme-mediated pathway.

The initiation of this loop begins with the multi-faceted habitat improvement orchestrated by the CBOFs. The porous, high-surface-area biochar matrix serves a dual purpose: it provides a critical physical refuge for microorganisms, creating protected microsites, and it directly immobilizes Sb and As through adsorption, surface complexation, and (co-)precipitation, thereby reducing their dissolved concentrations and acute toxicity (Hockaday et al., 2007; Xu et al., 2018). Simultaneously, the amendments function as a slow-release nutrient reservoir, supplying essential elements and labile organic carbon that stimulate microbial biomass growth and metabolic activity, providing the energy and building blocks for a more active community (Zhang et al., 2020).

Furthermore, the amendments induce comprehensive changes in the soil’s chemical environment. The inherent alkalinity of biochar typically raises soil pH, a shift that generally expands microbial diversity and can promote the transformation of Sb and As into more stable, less bioavailable solid-phase species (e.g., binding to Fe/Mn oxides, precipitation) (Rousk et al., 2010; Zhu et al., 2017). The concurrent increase in soil cation exchange capacity (CEC) enhances the retention of beneficial nutrient cations. These abiotic changes are complemented by the stimulation of key extracellular enzymes, as documented in Section 3.3, which reflects and accelerates the rates of core biogeochemical cycles (Laird et al., 2010).

This constellation of improved conditions—better habitat, reduced toxicity, ample nutrients, and stable chemistry—creates a new environmental filter that selectively enriches for a distinct, adapted, and functionally competent microbial consortium. This revitalized microbiome is not a passive bystander but an active participant in remediation. Based on the 16S rRNA sequencing data alone, we infer that the enriched Bacteroidota and Gemmatimonadota mainly contributed indirectly to plant tolerance by enhancing organic matter decomposition, nutrient cycling, and rhizosphere habitat quality, whereas their possible direct involvement in Sb/As transformation, such as As oxidation/reduction mediated by aioA- or ars-related genes, remains a hypothesis that requires further verification by functional gene quantification, metagenomics, or metatranscriptomics (Andres and Bertin, 2016; Li et al., 2024). The enriched microbial community likely contributes to long-term contaminant sequestration through various mechanisms: via bio-oxidation (e.g., oxidizing As(III) to As(V) or Sb(III) to Sb(V), which often adsorb more strongly), direct biosorption onto cell walls and exopolymers, and by facilitating secondary mineral precipitation. This biological activity establishes a positive feedback loop, further stabilizing contaminants and enhancing plant health through improved nutrient cycling and pathogen suppression.

In summary, the stabilization and remediation mechanism is an orchestrated, multi-stage process. Carbon-based organic fertilizers first directly intervene to immobilize contaminants and improve the soil’s physical–chemical matrix. This engineered environment selectively enriches for a beneficial and responsive microbial community. The metabolic activities of this community subsequently reinforce contaminant stabilization and support plant physiological health. The SEM analysis quantitatively corroborates this conceptual model, confirming that the increase in plant biomass is driven along two distinct pathways: an indirect route, where soil improvement reshapes the microbiome which then facilitates plant growth; and a direct route, where biochar rapidly elevates microbial functional activity, leading to enhanced plant performance. The ultimate remediation efficacy, therefore, emerges not from a single intervention but from the sophisticated synergy of chemical immobilization and microbiome-driven biological stabilization, elegantly orchestrated and statistically validated by the multifunctional properties of the applied amendments. It should be acknowledged that while the SEM analysis supports causal inference, the proposed mechanisms are inferred from measured parameters rather than directly observed at the molecular level. The enrichment of Bacteroidota and Gemmatimonadota is interpreted as indicative of enhanced nutrient cycling capacity, but direct evidence of their functional contributions—such as expression of genes involved in organic matter decomposition or metal(loid) transformation—remains to be established. Nevertheless, the integration of soil chemistry, enzymatic activity, microbial community analysis, and SEM provides a systems-level understanding of the remediation process that is consistent with the observed outcomes and supported by multiple lines of evidence.

## 4 Conclusions

This study demonstrates that engineered carbon-based organic fertilizers, particularly MgSO_4_-loaded biochar composites, provide an effective multi-mechanism strategy for remediating Sb-As co-contaminated agricultural soils. The key conclusions are as follows: First, different CBOFs exhibit distinct functional specificity—certain amendments such as hydrothermal carbon liquid optimize nitrogen and potassium availability, while the MgSO_4-_loaded biochar (CBOF-M) uniquely excels in enhancing soil organic carbon sequestration and reducing salinity. Second, the contrasting behaviors of Sb (immobilized) and As (mobilized) following the application of plain carbon amendments reveal a fundamental challenge in co-contaminated system remediation, underscoring the need for tailored amendment design that can simultaneously address both metalloids. Third, the most effective amendments, particularly CBOF-M, deliver a dual benefit by dramatically promoting crop growth while significantly reducing the translocation of toxic Sb to edible plant parts, thereby reconciling the often-conflicting goals of agricultural productivity and food safety. Fourth, successful remediation is mediated not only through direct physicochemical stabilization mechanisms but also via the amendment-induced targeted enrichment of beneficial rhizosphere microbial communities, including copiotrophic phyla such as Bacteroidota and Gemmatimonadota, which support long-term soil health and ecosystem functionality. Collectively, these findings establish that the remediation efficacy arises from an integrated mechanism wherein amendments simultaneously condition the soil habitat, restructure the microbial consortium, and enhance plant physiological performance. Therefore, the strategic design of multi-functional amendments, informed by site-specific contaminant interactions and soil properties, is essential for the sustainable and productive utilization of metalloid co-contaminated farmland. While this study provides robust correlational evidence and SEM-based causal inference for the proposed mechanisms, direct molecular-level validation—such as quantification of functional gene expression (e.g., arsenic oxidase, phosphatase-encoding genes) or isotope tracing of contaminant fluxes—would further strengthen the mechanistic understanding. Future studies employing metatranscriptomics and stable isotope probing are warranted to elucidate the specific microbial metabolic pathways contributing to contaminant stabilization and plant growth promotion.

## Data Availability

The data presented in this study are deposited in the National Microbiology Data Center (NMDC) repository under accession numbers NMDC40127377-NMDC40127383. The data are available at: https://nmdc.cn/resource/genomics/sra/detail/NMDC40127377, https://nmdc.cn/resource/genomics/sra/detail/NMDC40127378, https://nmdc.cn/resource/genomics/sra/detail/NMDC40127379, https://nmdc.cn/resource/genomics/sra/detail/NMDC40127380, https://nmdc.cn/resource/genomics/sra/detail/NMDC40127381, https://nmdc.cn/resource/genomics/sra/detail/NMDC40127382, https://nmdc.cn/resource/genomics/sra/detail/NMDC40127383.
